# A bacteriocin-based antimicrobial formulation to effectively disrupt the cell viability of methicillin-resistant *Staphylococcus aureus* (MRSA) biofilms

**DOI:** 10.1038/s41522-020-00166-4

**Published:** 2020-12-02

**Authors:** Christian Kranjec, Kirill V. Ovchinnikov, Torstein Grønseth, Kumar Ebineshan, Aparna Srikantam, Dzung B. Diep

**Affiliations:** 1 Faculty of Chemistry, Biotechnology and Food Science, Norwegian University of Life Sciences, Ås, Norway; 2 University of Oslo, Oslo, Norway; 3 Department of Otolaryngology, Head and Neck Surgery, Oslo University Hospital, Oslo, Norway; 4 Blue Peter Public Health and Research Centre, LEPRA Society, Hyderabad, India

**Keywords:** Antimicrobials, Biofilms, Microbiology

## Abstract

Antibiotic-resistant and biofilm-associated infections brought about by methicillin-resistant *Staphylococcus aureus* (MRSA) strains is a pressing issue both inside as well as outside nosocomial environments worldwide. Here, we show that a combination of two bacteriocins with distinct structural and functional characteristics, garvicin KS, and micrococcin P1, showed a synergetic antibacterial activity against biofilms produced in vitro by *S. aureus*, including several MRSA strains. In addition, this bacteriocin-based antimicrobial combination showed the ability to restore the sensitivity of the highly resilient MRSA strain ATCC 33591 to the β-lactam antibiotic penicillin G. By using a combination of bacterial cell metabolic assays, confocal and scanning electron microscopy, we show that the combination between garvicin KS, micrococcin P1, and penicillin G potently inhibit cell viability within *S. aureus* biofilms by causing severe cell damage. Together these data indicate that bacteriocins can be valuable therapeutic tools in the fight against biofilm-associated MRSA infections.

## Introduction

The genus *Staphylococcus* comprises a wide group of Gram-positive bacteria broadly distributed in the natural environments; representing one of the major commensal bacterial communities colonizing the skin and mucous membranes of humans and animals^1^. Among these, *Staphylococcus aureus* is considered to be a prominent opportunistic human pathogen, being associated with a range of clinical conditions: from self-remissive skin infections to life-threatening syndromes^2^. This coagulase-positive bacterium has traditionally been a problematic pathogen inside and outside the clinical setting; the main reason for this is its high propensity to acquire resistance to antibiotics^3–5^. In the last decades, however, the advent of community-associated (CA) MRSA strains significantly increased the incidence of staphylococcal antibiotic-resistant infections acquired outside the hospital environment. A range of skin and soft tissue infections account for the majority of CA-MRSA-associated syndromes; however, endocarditis and the involvement of pulmonary and osteoarticular sites, among others, have also been reported often as the secondary infection sites upon bacterial spread through the bloodstream^6–15^. A wide variety of mechanisms drive the acquisition of resistance to antimicrobials. Invariably, it involves the presence of genetic sequences mediating the resistance process; these can be intrinsically present within a bacterial population (i.e., ABC transporters) or inherited through the heterologous exchange of genetic material^16^. Of the latter mechanisms, one of the best described is the acquisition of the staphylococcal cassette chromosome *mec* (SCCmec) which confers resistance to β-lactams in MRSA strains^17,18^. In other cases, the decreased susceptibility to antimicrobials can be mediated in a bacterial population-based manner. For instance, the formation of biofilms represents a major therapeutic complication and, in some cases, makes the bacterial infection untreatable leaving the surgical removal of the infected area the sole therapeutic option^14^. Biofilms are produced upon switching of the bacterial growth mode from planktonic to sessile, where bacteria form multicellular aggregates embedded in an extracellular matrix-like substance. The formation of biofilms makes bacterial communities extremely refractory to hostile conditions; hence it is not surprising that biofilms are the prime mode of bacterial growth and colonization^19,20^. In the context of pathogenic bacteria, biofilms can form on biotic as well as abiotic surfaces, making the producing bacteria highly refractory to antibiotics and their infections persistent and recalcitrant^21,22^. It has been estimated that about 80% of all chronic infections are associated with biofilm formation^23^ and, among Gram-positive bacteria, *S. aureus* infections are among those with the highest association with biofilms^24,25^. Several mechanisms have been proposed for the decreased susceptibility to antimicrobials displayed by biofilms: including change in the pattern of bacterial gene expression, poor antimicrobial penetration, and reduction of the bacterial growth rate, among others^26–28^.

The combined effects of the emergence of antibiotic-resistant strains and their ability to form biofilms represent a serious threat in today’s medicine. In a recent statement the World Health Organization (WHO), indicated that the lack of investments and innovation in the development of novel antibiotics is undermining our ability to fight drug-resistant infections^29^, suggesting that the development of complementary antimicrobial strategies is of compelling importance. In recent years, the addressing of drug-resistance and biofilm formation mechanisms has led to the development of novel therapeutic strategies; where the treatment with traditional antibiotics is coupled with the use of different classes of antimicrobials and/or molecules able to interfere, at different levels, with the formation of biofilms^30,31^. Among these molecules, bacteriocins, ribosomally synthesized bacterial peptides, are a prime example^32^. It is thought that bacteriocins are produced virtually by all bacteria and, from an ecological point of view, bacteriocins are synthesized in order to confer a selective advantage to the producer in terms of niche colonization ability; since these molecules often display activity against closely related bacterial species^33,34^. Staphylococci are no exception with respect to bacteriocin production, and bacteriocins produced by several staphylococcal species have been shown to be active against pathogenic staphylococci, including *S. aureus*^35,36^. In the last decades, bacteriocins produced by lactic acid bacteria (LAB) have received great research attention. These microorganisms are common colonizers of dairy products, therefore LAB and the metabolites they produce are generally recognized as safe (GRAS) according to the Food and Drug Administration (FDA). With respect to this, two well-characterized LAB bacteriocins, nisin, and pediocin PA-1/AcH, have been approved for safe use as food bio-preservatives^37,38^. In addition, many LAB bacteriocins have been explored for medicinal applications since they have been shown to inhibit the growth of important human pathogens, including MRSA, vancomycin-resistant enterococci (VRE), and *Listeria monocytogenes*^39^. Recently, our group has conducted a survey of the microbial quality present in raw cow milk from farms in Kosovo; this work has led to the isolation of a large collection of LAB^40^. In turn, this allowed us to isolate and describe a novel group of leaderless bacteriocins, whose prototype member was named garvicin KS produced by a strain of *Lactococcus garvieae*^41^. Garvicin KS is a multi-peptide bacteriocin whose optimal activity depends on the action of three peptides in equimolar concentrations. In addition, this bacteriocin has been shown to have broad-spectrum antimicrobial activity and to synergize with nisin^41,42^. Here, using a combination of bacterial viability tests, confocal and electronic microscopy, we show that garvicin KS is able to inhibit the formation of *S. aureus* biofilms in vitro and to impair the bacterial viability in established biofilms. Moreover, in combination with a second bacteriocin, micrococcin P1, we demonstrate that garvicin KS is able to re-sensitize MRSA strains against penicillin G.

## Results

### The in vitro ability of garvicin KS to eradicate *S. aureus* biofilms is strain-dependent and is not augmented by micrococcin P1

Previous reports indicated that garvicin KS is effective in inhibiting the growth of *S. aureus* in liquid culture^42^, whereas micrococcin P1 has recently been regarded as a promising therapeutic alternative to antibiotics against MRSA and mycobacterial infections^43,44^. The antimicrobial potential of these bacteriocins, however, has never been assessed on clinically relevant *S. aureus* strains and, moreover, in a biofilm setting. In order to explore the antimicrobial activity of these two bacteriocins, we first tested their antimicrobial activity as well as the activity of 11 selected antibiotics against 6 *S. aureus* strains, all with a good biofilm-forming activity (Supplementary Fig. 1a). Among the strains, ATCC 10832, ATCC 29213, NEWMAN, and Sa3255 appeared sensitive to most antibiotics, 9 out of 11, 11 out of 11, 10 out of 11, and 10 out of 11, respectively. On the other hand, USA300 and ATCC 33591 appeared resistant to most antibiotics, 7 out of 11 and 8 out of 11, respectively. The antibiotic susceptibility test confirmed that two of these strains (USA300 and ATCC 33591) were resistant to cloxacillin, a chlorinated derivative of oxacillin, among other antibiotics, and therefore was designated as MRSA (Supplementary Table 1). Against planktonic cells, garvicin KS and micrococcin P1 displayed MIC_50_ values ranging from 25 to 50 μg/ml and 0.6 to 10 μg/ml, respectively (Table 1). It is interesting to note that the two bacteriocins synergized against the two MRSA strains, USA300 and ATCC 33591, among the six tested.

**Table 1:** MIC_50_ values for garvicin KS and micrococcin P1 determined in liquid culture for the indicated strains.

Antimicrobial (mg/ml)	Strain					
	ATCC 10832	Newman	USA 300	ATCC 29213	ATCC 33591	S.a. 3255
*Individual component*						
Garvicin KS	2.5 × 10^−2^	2.5 × 10^−2^	2.5 × 10^−2^	2.5 × 10^−2^	5 × 10^−2^	2.5 × 10^−2^
Micrococcin P1	6.3 × 10^−4^	3.13 × 10^−4^	6.3 × 10^−4^	2.5 × 10^−3^	>1 × 10^−2^	1 × 10^−2^
*Combination*						
Garvicin KS	6.3 × 10^−3^	3.13 × 10^−3^	1.56 × 10^−3^	1.3 × 10^−2^	1.3 × 10^−2^	1.3 × 10^−2^
Micrococcin P1	6.3 × 10^−4^	3.13 × 10^−4^	1.56 × 10^−4^	1.3 × 10^−3^	1.3 × 10^−3^	1.3 × 10^−3^
FIC^a^	1.25	1.13	0.31	1.04	0.39	0.65

^a^Synergy achieved with fractional inhibition concentration (FIC) ≤ 0.5.

**Fig. 1:**
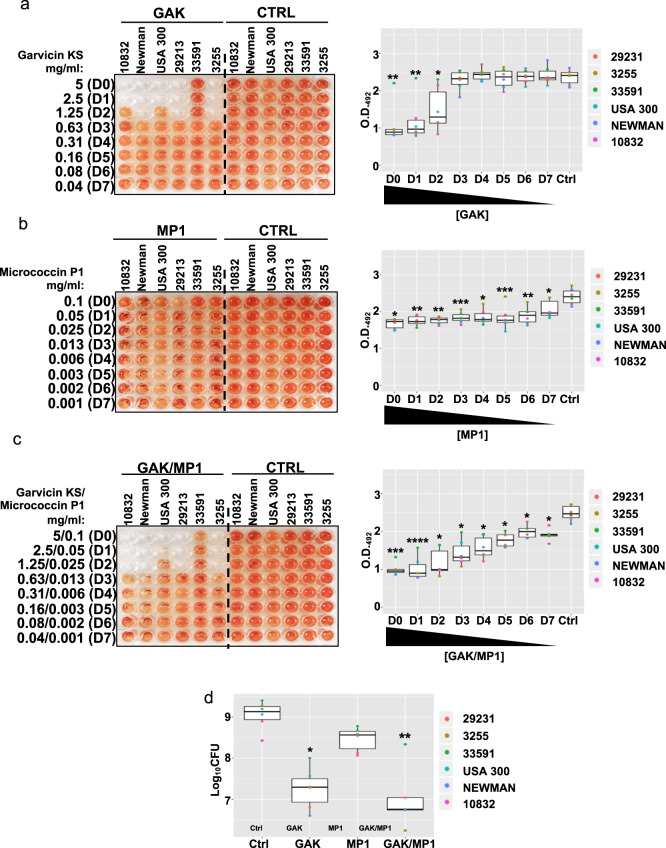
Assessment of garvicin KS and micrococcin P1 as individuals or in combination in eradicating *S. aureus* biofilms. The left panels show representative images of the BOAT assay performed with serial twofold dilutions (first six columns of the plate) of garvicin KS (**a**), micrococcin P1 (**b**) and both in combination (**c**) for the indicated strains. The concentration (in mg/ml) of the antimicrobials in the dilutions (dilution factors: D0–D7) is indicated on the far left of the images. At the same time, the assay was also performed using the control vehicles at their final concentrations (Ctrl, last six columns of each plate), see the “Methods” section for details on the composition. The development of red color indicates the retention of metabolic activity, and its quantification was performed by optical density readings at 492 nm (O.D. 492). The boxplots in the right panel show the trends of recovery of the bacterial metabolic activity as a function of the dilution factor for the different antimicrobials. Shown is the median distribution (thick line within boxes) and the degree of variability (amplitude of the box or interquartile region (IQR)) of the metabolic activities for the indicated strains measured at increasing dilution factors (D0–D7). Whiskers extending out the boxes mark the minimum and maximum observed values and the variability outside the middle 50% of values (whisker length). Outliers are represented as values that extend out of the whisker limit (1.5× IQR). Note that the combination treatment (GAK/MP1) led to a more delayed recovery of the bacterial metabolic activity compared to garvicin KS alone. **d** Boxplot showing the median distribution of logarithmic colony formation unit (Log_10_CFU) values calculated after the BOAT assay for the indicated strains. The concentrations used were 5 mg/ml for garvicin KS and 0.1 mg/ml for micrococcin P1. The data represent the average values obtained from three independent experiments. Asterisks above the boxplots represent the statistical significance (*p* value) as determined by Welch’s *t* test by comparing the median of each group with that of the respective control (Ctrl) group. Asterisk representation of statistical significance: *p ≤ 0.05; **p ≤ 0.01; ***p ≤ 0.001; ****p ≤ 0.0001.

It has been suggested that upon the formation of a biofilm, pathogens can become 10–1000 times less susceptible to antimicrobials^21,22^, we, therefore, used the MIC data produced for planktonic cells as a basis to set higher working concentrations to test on biofilms. These were 5 mg/ml for garvicin KS and 0.1 mg/ml for micrococcin P1. In order to test whether these two bacteriocins retained their antimicrobial effects on *S. aureus* biofilms, we used a modified version of the biofilm-oriented antimicrobial test (BOAT), where the metabolic activity indicator triphenyl-tetrazolium chloride was used to assess the susceptibility of the bacterial strains to the antimicrobials tested^45,46^. Biofilms from the selected *S. aureus* strains were allowed to form for 24 h before BOAT assays were performed using the bacteriocins indicated in Fig. 1 or their respective control (Ctrl) vehicles (see the figure legend and the “Methods” section). As can be observed, garvicin KS alone was sufficient to eradicate the biofilm-associated metabolic activity produced by five out of six *S. aureus* strains, including the MRSA strain USA 300, with MIC values ranging between 1.3 and 2.5 mg/ml (Fig. 1, Supplementary Fig. 2 and Table 2). Conversely, The *S. aureus* strain ATCC 33591 remained insensitive to the treatment, highlighting an over 100-fold MIC increase compared to the planktonic state (Table 2). Similarly, and in contrast with the results obtained from planktonic cultures (Table 1), the treatment with micrococcin P1 failed to abolish the biofilm-associated bacterial growth at concentrations up to 0.1 mg/ml and indeed resulted only in a weak, albeit significant, reduction of the median metabolic activities when compared with the control vehicle (Fig. 1b and Table 2). Interestingly, while the combination of the two bacteriocins failed to promote a synergistic effect for most strains, except for ATCC 10832 and Sa3255 (Table 2), it indeed brought about a steady and significant reduction of the metabolic activity for all tested strains (Fig. 1c and Supplementary Fig. 2).

**Table 2:** MIC_50_ values for garvicin KS and micrococcin P1 determined in biofilms for the indicated strains.

Antimicrobial (mg/ml)	Strain					
	ATCC 10832	Newman	USA 300	ATCC 29213	ATCC 33591	S.a. 3255
*Individual components*						
Garvicin KS	2.5	1.25	2.5	1.25	>5	1.25
Micrococcin P1	>1.0 × 10^−1^	>1.0 × 10^−1^	>1.0 × 10^−1^	>1.0 × 10^−1^	>1.0 × 10^−1^	>1.0 × 10^−1^
*Combination*						
Garvicin KS	1.25	1.25	5	1.25	>5	3.1×10^−1^
Micrococcin P1	2.5 × 10^−2^	2.5 × 10^−2^	1.0 × 10^−1^	2.5 × 10^−2^	>1.0 × 10^−1^	2.5 × 10^−2^
FIC^a^	0.75	1.25	1.5	1.25	ND^b^	0.5

^a^Synergy achieved with fractional inhibition concentration (FIC) ≤ 0.5.

^b^Not determined. The MIC values exceeded the maximum concentration tested: micrococcin P1, 1.0 × 10^−1^ mg/ml; garvicin KS, 5 mg/ml.

In order to monitor more directly the cell viability after the antimicrobial treatment, we performed a BOAT assay followed by colony-forming unit (CFU) counting. The treatment with garvicin KS and micrococcin P1 were repeated as above, but instead of adding the metabolic activity indicator, the remaining cells in the wells were serially diluted and spot-plated on agar dishes followed by incubation at 37 °C and CFU counting; the results of this assay are shown in Fig. 1d. As can be seen, and in line with the metabolic activity profiles, the treatments with garvicin KS (*p* = 0.022; Welch’s *t* test) and the combination garvicin KS/micrococcin P1 (*p* = 0.0013; Welch’s *t* test) led to a significant reduction in the median Log_10_CFU levels. Consistent with the results of the BOAT assays, the *S. aureus* ATCC 33,591, was the strain that retained the highest viability among those tested, representing the upper outlier for the combination treatment (Fig. 1d). In addition, it is interesting to note that although the metabolic activity of most of the strains was apparently abolished when garvicin KS was used, alone or in combination with micrococcin P1 at high concentrations (5 mg/ml), the CFU counting evidenced that variable levels of cell viability were indeed retained. This point will be further elaborated in the section “Discussion”.

Taken together, these data confirm previous studies that biofilms indeed form a protective environment for bacteria leading to a dramatic increase of the resistance to antimicrobials, and indicate that garvicin KS, alone or in combination with micrococcin P1, represents a potential antimicrobial compound alternative to antibiotics for the treatment biofilm-related *S. aureus* infections.

### The combined treatment garvicin KS and micrococcin P1 sensitizes MRSA strains against penicillin G

**Fig. 2:**
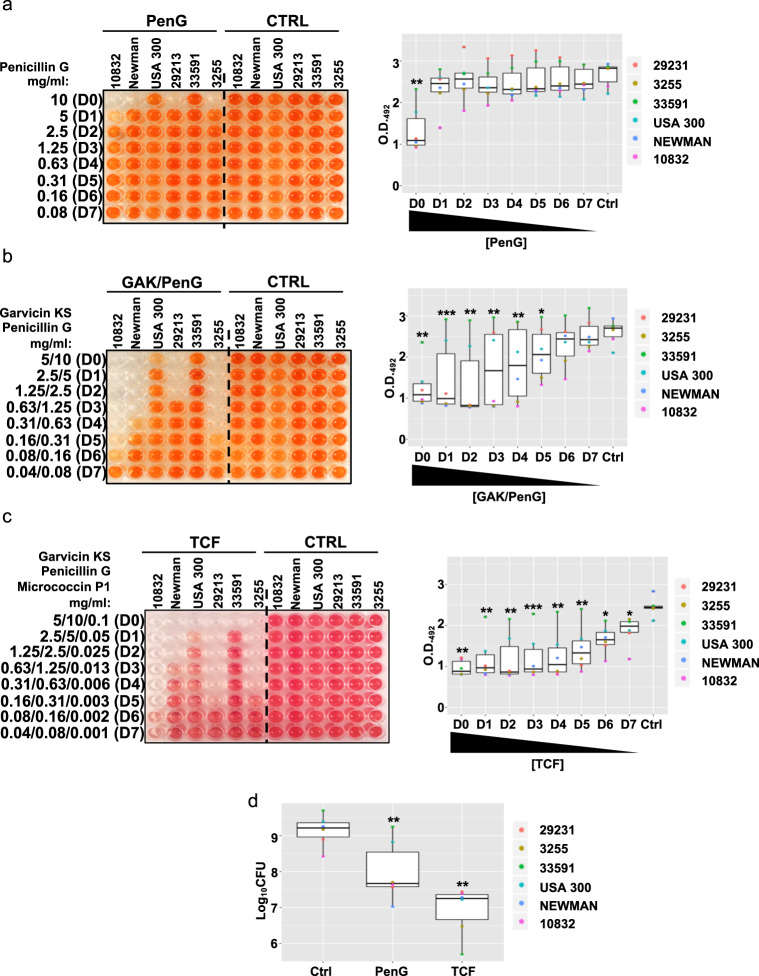
Garvicin KS and micrococcin P1 sensitize MRSA strains to penicillin G. **a** A representative image of the BOAT assay performed with penicillin G (Pen G, first six columns of the plate) or with its control vehicle (Ctrl, last six columns of the plate) is shown. The relative boxplot analyzing the trends of metabolic activity in the function of penicillin G dilution is shown on the far right. The BOAT assay and the relative metabolic activity quantifications were performed as detailed in Fig. 1. In **b** and **c**, similar experiments as in **a**, but the data refer to the combination treatment between garvicin KS and penicillin G (GAK/PenG), and the tricomponent formulation (TCF) between garvicin KS, micrococcin P1 and penicillin G, respectively. The control vehicles to their final concentrations were sterile distilled water in panel **a**, 0,02% (v/v) TFA in panel **b** and 0.033% (v/v) trifluoracetic acid/6.25% (v/v) 2-propanol in panel **c**. **d** Boxplot showing the median distribution of Log_10_CFU values obtained upon the indicated treatments and strains. The Log_10_CFU counting was performed following the BOAT assay for each indicated antimicrobial. The concentrations used for garvicin KS and micrococcin P1 were the same as in Fig. 1d, and the penicillin G concentration was 10 mg/ml. The control (Ctrl) samples were treated with an equivalent amount of the antimicrobial vehicles. All the data represent the average values obtained from three independent experiments. Asterisks above the boxplots represent the statistical significance (*p* value) as determined by Welch’s *t* test by comparing the median of each group with that of the respective control (Ctrl) group. Asterisk representation of statistical significance: *p ≤ 0.05; **p ≤ 0.01; ***p ≤ 0.001; ****p ≤ 0.0001.

Garvicin KS, like many other members of the leaderless bacteriocin group, possesses a net positive charge and an amphiphilic nature, which in turn allows it to interact with the prokaryotic membrane^47,48^. Although the mechanism of bacterial killing of garvicin KS has not been investigated in detail, it is reasonable to speculate that it follows a similar mode of action to other leaderless bacteriocins; that is, interacting with and destabilizing the bacterial membrane^47^. Previous studies have postulated that the treatment of bacteria with drugs that promote the destabilization of discrete membrane microdomains, known as lipid rafts, also lead to re-sensitization of resistant bacteria to antibiotics such as penicillins, due to the disruption of membrane-bound macromolecular complexes mediating the resistance process^49,50^. Based on this information, we reasoned that garvicin KS might lead to a similar effect generating a synergistic effect between the bacteriocin and penicillins. In order to test such a hypothesis, we repeated the BOAT assays to verify the degree of sensitivity of *S. aureus* biofilms to the β-lactamic antibiotic penicillin G, and subsequently tested the effects of the combination of the antibiotic with garvicin KS and micrococcin P1. The maximum concentration of penicillin G was set to 10 mg/ml considering the MIC values obtained for planktonic cells (Table 3). In line with the antibiotic sensitivity profiles shown in Supplementary Table 1, the treatment with penicillin G at the highest concentration (10 mg/ml; D0) led to growth inhibition and a reduction in the metabolic activity only for the non-MRSA strains (Fig. 2a, Supplementary Fig. 2 and Table 4). This further confirms that biofilm formation strongly diminishes the sensitivity of bacteria to antibiotics. On the other hand, the combination of penicillin G with garvicin KS produced a slower recovery of the metabolic activity, upon dilution of the antimicrobials, for the non-MRSA strains when compared to the antibiotic alone (Fig. 2b and Supplementary Fig. 2), which was also reflected by a reduction of the observed MIC values (Table 4). However, in contrast with what hypothesized, this combination failed to inhibit the growth of the two MRSA strains, USA300 and ATCC 33591. To search for the synergistic properties of micrococcin P1 with garvicin KS and penicillin G, we created a tricomponent formulation (TCF) containing all three antimicrobials. Indeed, this combination led to a further increase in antimicrobial activity, with a significant inhibitory effect on the metabolic activity of all strains (Fig. 2c, Supplementary Fig. 2 and Table 4). In line with these data, the BOAT assay followed by CFU counting confirmed that the TCF treatment led to a further reduction in the viability of the non-MRSA strains, and a significant reduction in the median Log_10_CFU values (*p* = 0,0027; Welch’s *t* test). This combination also led to a further reduction of the median Log_10_CFU values (*p* = 0,0013; Welch’s *t* test) and a more substantial inhibition of the viability of the MRSA strains (Fig. 2d). Furthermore, it is interesting to note that although the Log_10_CFU median values obtained upon the treatment with TCF were higher when compared with the garvicin KS/micrococcin P1 combination (Fig. 1d), albeit not significantly (*p* = 0.54; Welch’s *t* test); the treatment with TCF led to a strong and statistically significant reduction (*p* = 0.00067; Welch’s *t* test) of the viability of the MRSA strain ATCC 33591 (Fig. 2d), which was the most poorly affected strain by any other treatment.

**Table 3:** MIC_50_ values for penicillin G garvicin KS and micrococcin P1 determined in liquid culture for the indicated strains.

Antimicrobial (mg/ml)	Strain					
	ATCC 10832	Newman	USA 300	ATCC 29213	ATCC 33591	S.a. 3255
*Individual components*						
Penicillin G	<7.8 × 10^−3^	<7.8 × 10^−3^	>1	1.3 × 10^−1^	>1	<7.8 × 10^−3^
Combination						
Garvicin KS	<7.8 × 10^−4^	<7.8 × 10^−4^	2.5 × 10^−2^	1.3 × 10^−2^	5 × 10^−2^	<7.8 × 10^−4^
Penicillin G	<7.8 × 10^−3^	<7.8 × 10^−3^	2.5 × 10^−1^	1.3 × 10^−1^	5 × 10^−1^	<7.8 × 10^−3^
FIC^a^	ND^3^	ND^3^	1.25	1.52	1.5	ND^3^
*Combination*						
Garvicin KS	<7.8 × 10^−4^	<7.8 × 10^−4^	6.3 × 10^−3^	3.1 × 10^−3^	1.3 × 10^−2^	<7.8 × 10^−4^
Micrococcin P1	<7.8 × 10^−5^	<7.8 × 10^−5^	6.3 × 10^−4^	3.1 × 10^−4^	1.3 × 10^−3^	<7.8 × 10^−5^
Penicillin G	<7.8 × 10^−3^	<7.8 × 10^−3^	6.3 × 10^−2^	3.1 × 10^−2^	1.3 × 10^−1^	<7.8 × 10^−3^
FIC^b^	ND^3^	ND^3^	1.32	0.49	0.52	ND^c^

^a^Synergy achieved with fractional inhibition concentration (FIC) ≤ 0.5, for two components.

^b^Synergy achieved with fractional inhibition concentration (FIC) ≤ 0.75, for three components.

^c^Not determined. The MIC values exceeded the minimum concentration tested: penicillin G, 7.8 × 10^−3^ mg/ml; micrococcin P1, 7.8 × 10^−^^5^ mg/ml; garvicin KS 7.8 × 10^−4^ mg/ml.

**Table 4:** MIC_50_ values for penicillin G garvicin KS and micrococcin P1 determined in biofilms for the indicated strains.

Antimicrobial (mg/ml)	Strain					
	ATCC 10832	Newman	USA 300	ATCC 29213	ATCC 33591	S.a. 3255
*Individual components*						
Penicillin G	10	10	>10	10	>10	10
*Combination*						
Garvicin KS	3.1 × 10^−1^	6.3 × 10^−1^	>5	1.25	>5	3.1 × 10^−1^
Penicillin G	6.3 × 10^−1^	1.3	>10	2.5	>10	6.3 × 10^−1^
FIC^a^	0.12	0.63	ND^c^	1.25	ND^c^	0.31
*Combination*						
Garvicin KS	1.6 × 10^−1^	1.25	5	3.1 × 10^−1^	5	3.1 × 10^−1^
Micrococcin P1	3.1 × 10^−3^	2.5 × 10^−2^	1 × 10^−1^	6.3 × 10^−3^	1 × 10^−1^	6.3 × 10^−3^
Penicillin G	3.1 × 10^−1^	2.5	10	6.3 × 10^−1^	10	6.3 × 10^−1^
FIC^b^	0.12	1.5	ND^c^	0.41	ND^c^	0.41

^a^Synergy with fractional inhibition concentration (FIC) ≤ 0.5, for two components.

^b^Synergy with fractional inhibition concentration (FIC) ≤ 0.75, for three components.

^c^Not determined. The MIC values exceeded the maximum concentration tested: penicillin G, 10 mg/ml; micrococcin P1 1.0 × 10^−1^ mg/ml; garvicin KS 5 mg/ml.

**Fig. 3:**
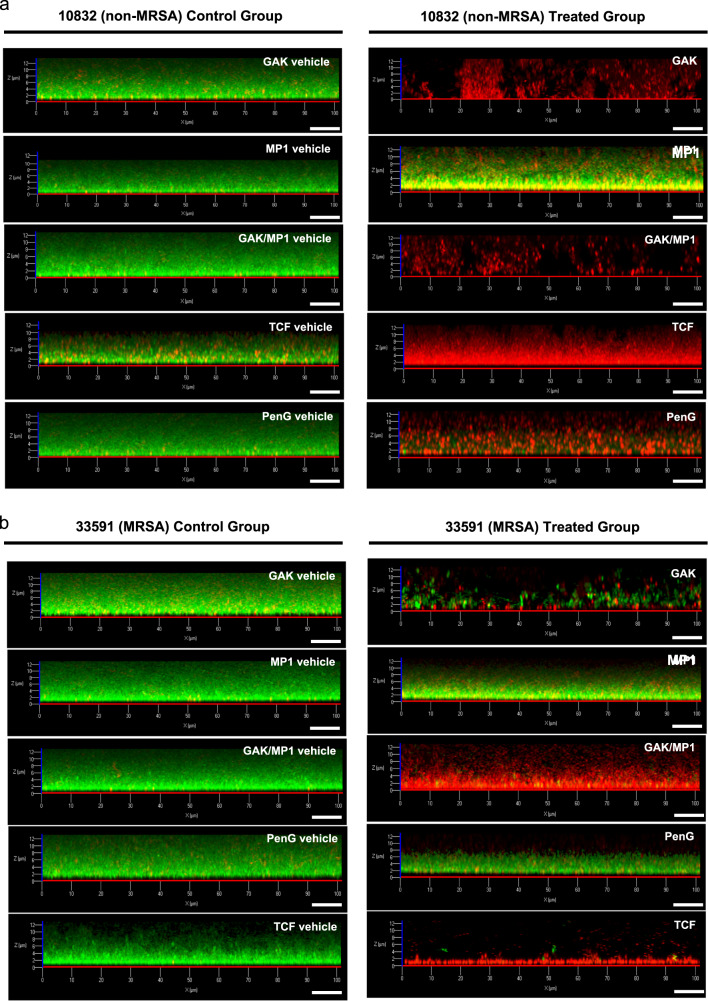
Confocal laser scanning microscope images of *S. aureus* biofilms exposed to antimicrobial treatments. **a** Biofilms for the ATCC 10832 (non-MRSA) strain was allowed to form on glass-bottomed chambers for 24 h prior to be treated with the indicated antimicrobials or their combinations (right set of panels) or the respective control-vehicles (left set of panels). The biofilms were subsequently stained using the LIVE/DEAD biofilm staining kit and confocal microscope images were taken using a 63× oil immersion objective. **b** Same as in **a** with the exception that the strain used was the MRSA strain ATCC 33591. Scale bars correspond to 10 μm. The control vehicles were the same as described in Figs. 1 and 2.

Next, we were interested in performing a more in-depth visual investigation of the biofilms produced in vitro and of the effects elicited by the treatments described above within the biofilms. In order to address this point, we performed a LIVE/DEAD biofilm staining followed by confocal microscopy analysis by using a combination of two fluorophores: SYTO-9 and propidium iodide (PI), which are known to selectively stain live and dead bacterial cells, respectively^45,51^. The strains ATCC 10832 and ATCC 33591 were chosen for this analysis as representatives of a methicillin-sensitive and -resistant strains, respectively; the results of these experiments are shown in Fig. 3. As can be seen, the control vehicle (Ctrl)-treated samples produced biofilms with a thickness up to 12 μm and largely dominated by SYTO-9-positive (green—live) bacterial cells for both tested strains (Fig. 3a, b, left set of panels). In good agreement with the results presented above, the treatment with the antimicrobials described in Figs. 1 and 2, produced strain-dependent effects. For ATCC 10832, with the exception of micrococcin P1, all the treatments promoted a dramatic shift in the staining pattern; with a strong increase in the proportion of PI-positive (red—dead) cells (Fig. 3a, the right set of panels). The ATCC 33591 strain, on the other hand, showed an increased resistance towards the treatment with garvicin KS and penicillin G, whereas the combined treatments with garvicin KS/micrococcin P1 (GAK/MP1) and particularly with the TCF, strongly impacted on cell viability (Fig. 3b, the right set of panels).

Taken together, these results indicate that the combined treatment with garvicin KS and micrococcin P1 sensitizes MRSA strains to penicillin G. Furthermore, these results also demonstrate that the use of a combinatory formulation between bacteriocins and antibiotics produce a strong growth-inhibitory and synergistic effect against multidrug-resistant *S. aureus* strains as demonstrated here with the MRSA strains USA300 and ATCC 33591.

### The formulation and its components are effective in abolishing the growth of *S. aureus* clinical strains

**Fig. 4:**
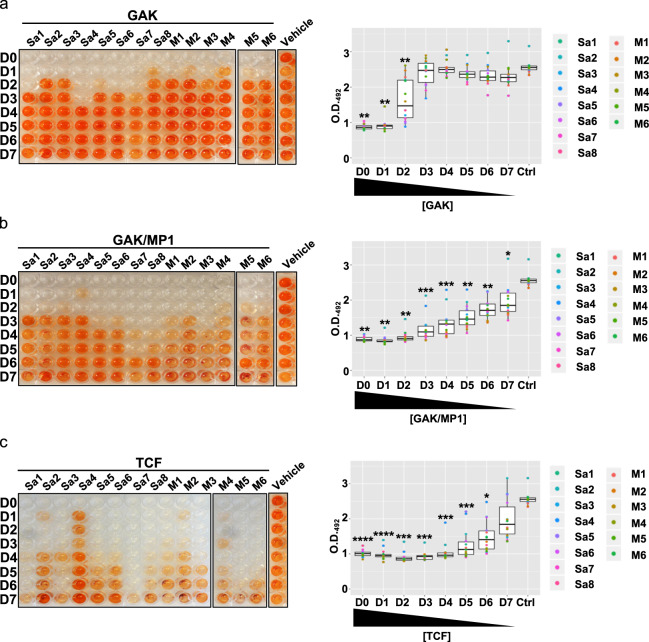
Sensitivity of the clinical *S. aureus* strains to garvicin KS alone, in combination with micrococcin P1, and to the tricomponent formulation. The indicated strains were treated with garvicin KS (**a**), micrococcin P1 (**b**), or the tricomponent formulation (**c**). Representative images of the BOAT assay performed are shown in the left panel while the corresponding boxplots analyzing the trend of metabolic activity recovery are shown in the right panel. The assays were performed on eight methicillin-sensitive (Sa1–8) and on six methicillin-resistant (M1–6) strains. In the vehicle columns, the strain MRSA6 (M6) was used as a representative of the outcome for all vehicle-treated strains. The BOAT assays, antimicrobial vehicles, and the relative quantifications were performed as indicated in Figs. 1 and 2. All the data represent the average values obtained from three independent experiments. For the control vehicles of the antimicrobials see the legend in Fig. 2 and the “Methods” section. Asterisks above the boxplots represent the statistical significance (*p* value) as determined by Welch’s *t* test by comparing the median of each group with that of the respective control (Ctrl) group. Asterisk representation of statistical significance: *p ≤ 0.05; **p ≤ 0.01; ***p ≤ 0.001; ****p ≤ 0.0001.

Having found that garvicin KS, either alone or in combination with micrococcin P1 and penicillin G, is effective in eradicating the biofilm-producing *S. aureus* strains, we moved on assessing the antimicrobial effects of these combinations on *S. aureus* strains derived from nosocomial skin infections. To this end, we obtained a panel of bacterial strains isolated from skin ulcers due to secondary infections in leprosy patients from the Blue Peter research Centre in India (Supplementary Fig. 3a). A total of 31 strains were obtained, of which 14 were confirmed to be *S. aureus* isolates: all displayed a good biofilm formation ability in vitro (Supplementary Fig. 3b) and, among these, six strains were confirmed MRSA (Supplementary Table 2). When we repeated the BOAT assay on biofilms produced with the clinical isolates, garvicin KS alone showed significant antimicrobial activity against all the strains (Fig. 4a and Supplementary Fig. 4) albeit at low dilution factors, with a pattern of recovery of the metabolic activity that resembled that obtained in Fig. 1a (Fig. 4a, right panel). In good agreement with the data presented above, the addition of micrococcin P1 and penicillin G further increased the efficacy of the formulation and progressively extended the range of dilution factors that retained antimicrobial activity (Fig. 4b, c and Supplementary Fig. 4).

### The tricomponent formulation causes visible cell damage in treated staphylococcal biofilms

**Fig. 5:**
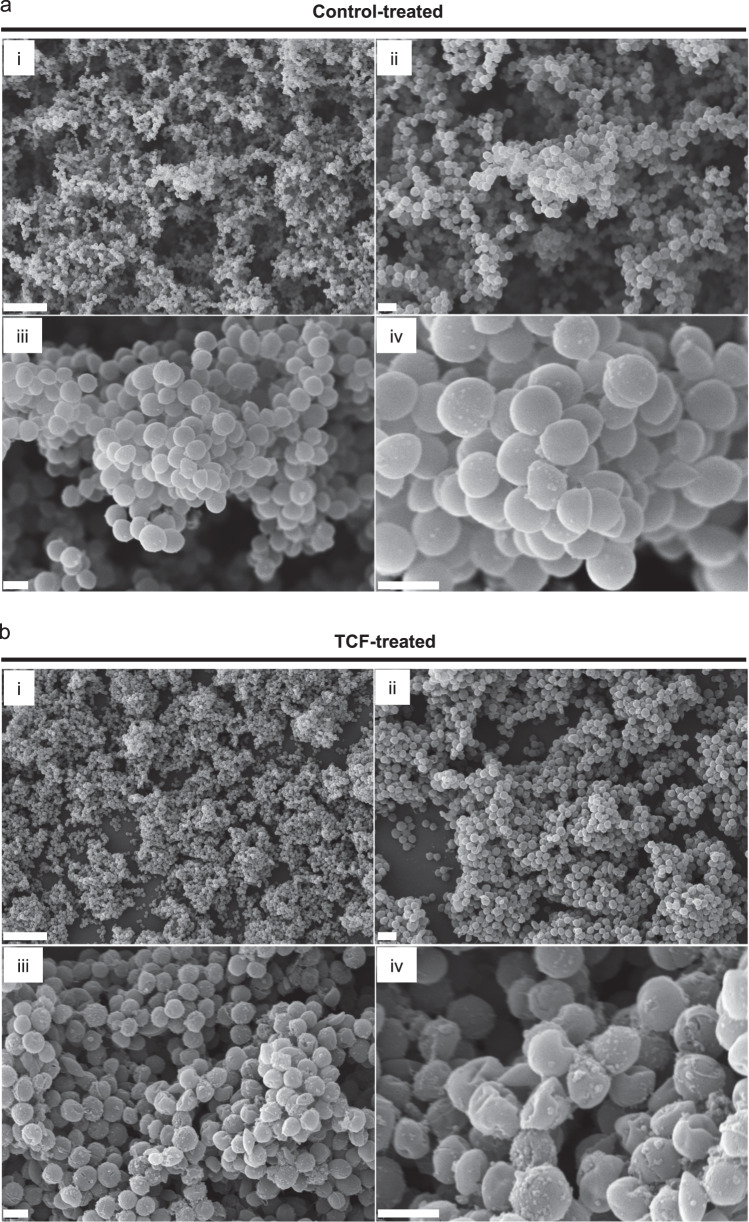
Visualization of the effects of the tricomponent formulation on *S. aureus* biofilm-associated cells. Biofilms were treated with the TCF control vehicle (**a**) or the tricomponent formulation (**b**) and then subjected to scanning electron microscopy (i, 3500×; ii, 7500×; iii, 20,000×; iv, 50,000×). Scale bars in the subpanels i–iv are 10, 2, 1, and 1 μm, respectively.

To further characterize the effect of the TCF, *S. aureus* biofilms were allowed to develop on the surface of glass slides and then treated for 24 h with the TCF and the vehicle as control. The morphology of biofilm architecture was then analyzed using scanning electron microscopy (SEM) and the results are shown in Fig. 5. As can be seen in Fig. 5a, and consistent with previous studies, the control-treated biofilm appeared as large and multilayered aggregates of cells with interspersed extracellular polymeric substance surrounding the cell clusters^52–54^. Conversely, at low magnifications the TCF-treated biofilm appeared to have a lower cell density (and a reduced thickness) compared to the control, as larger areas of the underlying glass surface could be observed (Fig. 5b, upper panels). At higher magnifications, it became apparent that cells undergone severe damage as their morphology appeared irregular and deformed compared to control-treated cells (Fig. 5b, lower panels). In addition, the TCF-treatment also produced a significant amount of larger particles on the surface of the cells, which we speculate to be cell contents or debris from damaged cells.

## Discussion

In this study, we report the use of a TCF based on the use of the bacteriocins garvicin KS and micrococcin P1 along with penicillin G as an eradication agent against *S. aureus* biofilms produced in vitro. In particular, we show that the combination between the two bacteriocins is able to sensitize MRSA strains against penicillin G and to potentiate its overall antimicrobial action. Importantly, garvicin KS, and its antimicrobial combinations with micrococcin P1 and penicillin G, have been shown to be highly effective on biofilms produced by clinical *S. aureus* strains, including several MRSA, isolated from plantar skin ulcers from leprosy-afflicted patients. Garvicin KS and micrococcin P1 are structurally and functionally distinct. The former is a multi-peptide bacteriocin composed of three nonmodified peptides and belongs to the leaderless family of bacteriocins (class IId)^41^. Its mode of killing has not been elucidated, however, it is thought to disrupt the membrane integrity leading to leakage of extracellular fluids subsequent lysis and cell death (unpublished data). Conversely, micrococcin P1 belongs to a class of microbial ribosomally synthesized and post-translationally modified peptides^55^ known as thiopeptides; a group of potent inhibitors of protein synthesis^56–59^.

In order to assess the effects of the formulation, and of its single components, on *S. aureus* biofilms, we have monitored the residual metabolic activity after each treatment by using an adapted version of the BOAT assay^45^. It is interesting to note that although garvicin KS was relatively effective at inhibiting the growth of all the tested strains in their planktonic growth mode (MIC_50_ between 2.5 × 10^−2^ and 5.0 × 10^−2^ mg/ml), biofilms produced by the MRSA strain ATCC 33591 showed increased resilience to the treatment. This highlights the fact that, in our hands as in others, the biofilm formation confers *S. aureus* the ability to tolerate antimicrobial concentrations at least 50–100 times higher when compared with the planktonic condition. In this context, previous hypotheses suggested that a poor penetration of the antimicrobials within the biofilm could be, at least in part, the reason behind the increased resilience to antimicrobial therapies^60^. Our confocal microscopy analysis, however, suggests that a drastic reduction in penetration of the antimicrobials is unlikely. Treatments with garvicin KS alone or in combination with micrococcin P1 and penicillin G differentially led to a consistent and uniform switch from SYTO-9 to PI positivity within the biofilms produced by the strains ATCC 10832 and ATCC 33591. Although we did not verify additional influencing factors, other studies indicated that modulation in the pattern of microbial gene expression is likely to be a key factor in conferring antimicrobial resistance to biofilms^21,61^.

Similarly to garvicin KS, micrococcin P1 also showed potent activity against most of *S. aureus* strains when grown in planktonic conditions (MIC_50_ between 6.3 × 10^−4^ and 1.0 × 10^−2^ mg/ml); conversely, however, micrococcin P1 failed to abolish the metabolic activity in biofilms produced by the same strains, even at concentrations that exceeded 100 times the planktonic MIC. An interesting observation highlighted by our study was the ability of bacteriocins to cooperate with penicillin G and, importantly, to sensitize the highly resilient ATCC 33591 MRSA strain to the antibiotic. The use of penicillins has declined over the second half of the 19th century due to the rapid spread of resistance among pathogenic bacteria^62^. The cooperative effect with bacteriocins could therefore rehabilitate the therapeutic use of β-lactam antibiotics to treat MRSA-induced infections over more costly and toxic alternatives. The conditional loss of resistance to β-lactams by MRSA strains has been reported previously^63^. These studies indicated that the perturbation of the penicillin-binding protein 2a in connection to an alteration of the plasma-membrane function were responsible for this effect. In our study we found that garvicin KS alone was ineffective to re-sensitize *S. aureus* USA 300 and ATCC 33591, the two MRSA strains, to penicillin G; and it indeed required the combination with micrococcin P1. The research aimed at exploring the use of micrococcin P1 into drug formulations has not substantially progressed until recent years due to its poor water-solubility and the fact that its biosynthetic pathway has been elucidated only recently^64^, therefore little is known about what additional and combinatorial effects micrococcin P1 could mediate to promote the re-establishment of penicillin sensitivity.

When the bacterial viability in treated biofilms was assessed by CFU counting, an apparent discrepancy with the results from the BOAT assays was observed. Indeed, treatments that led to the abolishment of metabolic activity did not translate directly into a corresponding annihilation of the cell viability when quantified by CFU counting. A possible explanation for such an effect can be offered by biofilm-associated cell dormancy. Bacterial dormancy is a well-documented phenomenon and an active field of research in bacterial biofilms, and it significantly contributes to the long-term persistence of bacterial cells^65,66^. The dormant phenotype is characterized by low levels of metabolic activity, which confers the reduced susceptibility to antimicrobials^66,67^. It is therefore plausible to speculate that if persister cells do exist within the biofilms produced in our model, their metabolic activity might not be tracked in the BOAT assays; however, metabolic reactivation may occur once cells are placed in an appropriate growth-promoting environment^68,69^.

Our BOAT analysis on biofilms produced by clinical *S. aureus* strains largely confirmed the results described above. According to the analysis of microbial diversity in secondary skin infections in leprosy patients, *S. aureus* was one of the main pathogens responsible for the colonization of these sites. The prevalence of MRSA strains in infected wounds has been found to be between 50 and 60% in India and other parts of the world^70^. Consistent with this, about 50% of the *S. aureus* strains obtained from India were classified as MRSA. Interestingly, all the clinical *S. aureus* isolates, including the MRSA strains, appeared to be highly sensitive to our treatments (see Fig. 4). In particular, the TCF appeared to have the strongest effect, however, also garvicin KS alone or in combination with micrococcin P1 showed the ability to inhibit the metabolic activity of all the strains.

This study indicates that garvicin KS and micrococcin P1 have the potential to be used as anti-biofilm agents. In addition, this work highlights the antimicrobial properties of bacteriocins and suggests that their development as therapeutic tools, possibly in combination with antibiotics, could provide future options to treat bacterial infections associated with antibiotic resistance and biofilms.

## Methods

### Bacterial strains and growth conditions

The strains ATCC 10832, ATCC 29213, ATCC 33591, and *S. aureus* Newman (D2C—ATCC 25904) were purchased from ATCC and have been described previously^71–73^. The methicillin-sensitive *S. aureus* strain ATCC 29213 was also purchased from ATCC and has been previously used as a reference strain for the determination of performance standards for antimicrobial disk susceptibility tests by the Clinical and Laboratory Standards Institute (CLSI) (see the ATCC website for more details). The community-acquired MRSA USA300 (strain JE-2) was a gift from Prof. Morten Kjos, Molecular Microbiology Research Group, Norwegian University of life Sciences, Norway and the methicillin-sensitive *S. aureus* strain 3255 was a kind gift from Prof. Hans-Georg Sahl, Medical Microbiology Group, University of Bonn, Germany. The clinical *S. aureus* strains were obtained from Blue Peter Public Health and Research Centre (BPHRC), LEPRA Society, Hyderabad, India and were isolated from plantar ulcers of patients suffering from leprosy. Leprosy patients with plantar ulcers who were registered at LEPRA Society BPHRC, from December 2018 through May 2019 (*n* = 31) were enrolled in the study after obtaining written informed consent. The study protocol was approved by the Institutional Ethical Committee at LEPRA Society and complied with all ethical requirements for the proposed research. When photographs of the plantar ulcers were taken, written consent for their publication was obtained from the patients. The protocol was aimed at isolating prevalent bacterial species responsible for plantar ulcer infections. A total of 31 wound swabs were collected using a sterile pure viscose swab (Hi-media, Mumbai, India) and transported immediately for further processing to the microbiology laboratory. One swab was collected from each leprosy foot ulcer. Each swab was inoculated on Blood and *McConkey* agar plates and bacterial isolates were allowed to grow overnight at 37 °C. Cells were identified by colony morphology, Gram staining and other standard tests^74^. In addition, all isolates were subjected to species-level identification by matrix-assisted laser desorption/ionization-time of flight mass spectrometry (Vitek MS system, bioMerieux, France) as per the manufacturer’s instructions. The identity of the bacterial species was further confirmed by 16S rRNA genotyping. All wound swabs yielded mono-bacterial culture. Out of 31 isolates, the majority of the species identified, 45.16% (14/31), were confirmed to be *S. aureus* isolates and all were biofilm producers. Additional bacterial species were *S. haemolyticus*, *Corynebacterium striatum*, *Proteus spp*., *Moraxella spp*., *Enterococcus faecalis*, *Pseudomonas spp*., *Citrobacter koseri*. All *S. aureus* strains were grown O/N in tryptic soy broth (TSB) (Sigma) at 37 °C in aerobic conditions without shaking.

### Antimicrobials and vehicles

Garvicin KS peptides, (GAK-A, GAK-B, and GAK-C) were synthesized by Pepmic Co., Ltd., China with 90–99% purity. Micrococcin P1 was purchased from Cayman Chemical, Michigan, USA with ≥95% purity. Garvicin KS was solubilized to concentrations of 10–30 mg/ml in 0.1% trifluoracetic acid (TFA) (Sigma). Micrococcin P1 was solubilized in a 50% (v/v) mixture of isopropanol (Merck) with 0.1% (v/v) TFA. Penicillin G (Sigma) was solubilized in sterile distilled water to a stock concentration of 100 mg/ml and further sterilized by microfiltration through a filter with a pore size of 0.45 μm (Sarstedt). Antibiotic discs were from Oxoid. All antimicrobials and antibiotics were stored at −20 °C until use.

#### Planktonic cell growth inhibition assays and antimicrobial synergy determination

Growth inhibition assays were performed in 96-well microtiter plates as described previously^42^. Briefly, 135 μl of TSB were dispensed in each well of a microtiter plate (according to the number of bacterial strains tested) except in the wells of the first row. The antimicrobials were diluted in TSB to working concentrations in a final volume of 285 μl and dispensed in the wells of the first row. The working concentrations were 100 μg/μl for garvicin KS, 10 μg/μl for micrococcin P1, and 1 mg/ml for penicillin G. From the first row, 150 μl of the antimicrobials were then serially diluted (twofold dilutions) in a sequential fashion until the last row of the plate. Finally, 15 μl of a fresh O/N culture of each strain were added in the appropriate wells to reach a final volume of 150 μl in each well. The plates were then incubated at 37 °C for 24 h. The growth inhibition was expressed as a minimum inhibitory concentration (MIC_50_), which refers to the minimum concentration of the antimicrobial needed to reduce at least 50% of the microbial growth compared to the untreated control. The MIC_50_ was assessed by optical density readings at 600 nm (O.D._600_).

Synergistic interactions between antimicrobials were determined using the fractional inhibition concentration (FIC). The FIC values were calculated as follows: FIC = FICa + FICb + FICc, where the FICa means MIC of A in combination/MIC of A alone, FICb means MIC of B in combination/MIC of B, and FICc means MIC of C in combination/MIC of C alone. Effects were considered as synergistic if FIC was ≤0.5 for two components mixture^75^ and ≤0.75 for three components mixture^76^.

### Antibiotic susceptibility testing (AST)

For all ATCC *S. aureus* strains and the strains USA300 and 3255, AST was assessed using the Kirby–Bauer disc diffusion susceptibility protocol^77^. One day prior to the inoculum preparation, the microorganisms were subcultured. Using a sterile inoculating loop, five well‐defined colonies were touched and suspended in 5 mL of sterile BHI broth and incubated until a cell density equal to 0.5 McFarland standard (∼1 × 10^8^ CFU/mL) was achieved. The inoculum was then spread on the surface of Mueller–Hinton agar plates. Agar plates were left to dry in a sterile hood for 10–15 min before antibiotic discs (Oxoid) were applied with a disc dispenser (Oxoid). The inhibition zones were evaluated after a 24 h incubation at 37 °C by recording the zone diameter and antibiotic susceptibility scoring was attributed based on the CLSI interpretative standards.

For all clinical *S. aureus* strains obtained from India, AST was carried out on VITEK-2 system, according to the manufacturer’s (bioMerieux, France) instructions, by using pure overnight subcultures. Briefly, 3–5 colonies from a pure bacterial culture which has similar morphology were transferred into 3.0 mL of sterile saline (0.50% NaCl in H_2_O) by using sterile swab in a 12 × 75 mm clear plastic (polystyrene) test tube. Then, turbidity was adjusted to McFarland range 0.50–0.63 for isolates using a DensiChek^TM^. The inoculum was dispensed into reagent cards placed in a cassette consisting of antimicrobials and manually placed into the incubation chamber. Inoculated cards were passed by a mechanism, which cut off the transfer tube and sealed the card prior to loading into the carrousel incubator. All cards were incubated at 37 °C for growth and optical densities at 600 nm were read once every 15 min during the entire incubation period. The VITEK-2 system analyzed the data and determined the sensitivity. The data were automatically recorded and the sensitivity pattern was generated by the VITEK-2 Compact Software in the computer which prints the sensitivity pattern of the test organism. Each isolate was interpreted as sensitive, intermediate, or resistant for each drug tested as recommended by the manufacturer (bioMerieux, France) instructions (bioMerieux, France).

### In vitro biofilm production and biofilm formation ability assay

*S. aureus* strains were inoculated in 5 ml of TSB and incubated O/N at 37 °C. Ten microlitres of the O/N cultures were then inoculated in 90 μl of TSB supplemented with 1% glucose and 1% NaCl (TSB-GN) in the appropriate wells of a 96-well microtiter plate (Sarstedt) to a final volume of 100 μl. The plates were then incubated at 37 °C for 24 h. After the incubation, the presence of the biofilm at the bottom of the wells was assessed visually.

Biofilm formation ability assays were performed as described previously^78^ with some modifications. *S. aureus* biofilms were allowed to form for 24 h prior to being washed twice with 100 μl of 0.9% NaCl saline buffer at room temperature (RT) to remove the planktonic cells and left to dry for 15 min. After drying, 200 μl of a 0.4% solution of crystal violet (Sigma) were added to each well and incubated for an additional 15 min. The dye was then removed and the wells were washed three times with 200 μl of 0.9% NaCl saline buffer, and the biofilm-bound crystal violet was then extracted by incubating the wells with 100 μl 70% ethanol. The extraction procedure was repeated twice and the combined crystal violet amount extracted was quantified by O.D. reading at 600 nm. The quantification of the crystal violet released from the biofilm is a surrogate measure of the number of bacterial cells forming the biofilm. Blank controls consisted of 150 μl of plain TSB-GN.

### Biofilm-oriented antimicrobial test (BOAT)

The BOAT assay was performed as described previously^45^ with some modifications. The serial dilutions of the antimicrobials were prepared in challenge plates as follows: 175 μl of TSB were transferred in each row of a 96-well microtiter plate, except for the first row, according to the number of microbial strains to treat. In the first row of the plate, the antimicrobials were diluted to their respective working concentrations in a final volume of 350 μl of TSB. From the first row, 175 μl of the antimicrobial dilutions were then transferred to the second row of the plate and further serially diluted to the bottom of the plate. The same procedure was followed to prepare the controls, except that instead of the antimicrobials an equivalent volume of the respective vehicles was used. Unless otherwise stated, the starting concentrations of the antimicrobials for all experiments involving biofilms were 5 mg/ml for garvicin KS, 0.1 mg/ml for micrococcin P1, and 10 mg/ml for penicillin G. At the same time, the assay was also performed using the control vehicles to their working concentrations: 0.02% (v/v) TFA for garvicin KS, 0.013% (v/v) TFA/6.25% (v/v) 2-propanol for micrococcin P1 and the mixture of 0.033% (v/v) TFA/6.25% (v/v) 2-propanol for the combination of the two and the TCF. The control vehicle for penicillin G was sterile distilled water. The biofilms were allowed to form for 24 h and then washed twice with 100 μl of sterile saline buffer and a total of 150 μl of the antimicrobial and control dilutions were transferred from the challenge plate to the corresponding wells of the biofilm plate. The challenged biofilms were then incubated for an additional 24 h at 37 °C. After the challenge period, the antimicrobial dilutions were removed and the biofilms were carefully washed three times with 150 μl of the sterile saline buffer. A total of 100 μl of TSB supplemented with 0.025% of triphenyl-tetrazolium chloride (TTC, Sigma) were then added to each well of the plate and further incubated at 37 °C for 5 h. The results were then assessed by monitoring the development (or not) of red formazan (red color), denoting the retainment of metabolic activity by bacterial cells^46,79^. The medium was then removed and 200 μl of ethanol:acetone (70:30) mixture was added to the wells and incubated O/N in order to extract the red formazan. The amount of extracted dye, reflecting the degree of bacterial cell metabolic activity, was then quantified by spectrophotometric readings at 492 nm. The metabolic activity inhibition for biofilm-associated cells was expressed as MIC_50_, which refers to the minimum concentration of the antimicrobial needed to reduce at least 50% of the metabolic activity compared to the untreated control. The MIC_50_ was assessed by optical density readings at 492 nm (O.D._492_) upon solubilization of the metabolic activity indicator TTC.

### Determination of the bacterial viability after BOAT

The procedure for the BOAT assay was repeated as described above except that instead of adding the TTC solution, the antimicrobial-challenged cells were resuspended in TSB and then serially diluted in TSB buffer. Serial dilutions of the bacterial cells were then plated on BHI agar plates and incubated at 37 °C for 24 h. The results were then assessed by direct counting of the developed colonies and the CFU was determined.

### Laser scanning confocal microscopy

Biofilms were allowed to form for 24 h as described above with the exception that they were formed in the wells of chambered cover-glass plates (Thermo Fisher Scientific) before being challenged with the antimicrobials or the respective control vehicles diluted in TSB. Biofilms were then treated with the LIVE/DEAD Biofilm Viability Kit (Molecular Probes, Thermo Fisher Scientific) according to the manufacturer´s instructions. Z-stacks of the stained biofilms were then taken on a confocal laser scanning microscope (Zeiss), using a 488 nm argon laser line for exciting the SYTO-9 (green) dye and a 561 nm laser line for the PI (red).

### Scanning electron microscopy

For this analysis, *S. aureus* biofilms were grown on 8 mm rounded glass coverslips for 24 h before being challenged with the antimicrobials or the respective control vehicles diluted in TSB. After the antimicrobial treatment, the biofilms were carefully washed twice in phosphate-buffered saline and then fixed in 3% glutaraldehyde O/N. Subsequently, biofilms were subjected to dehydration in increasing alcohol series of 30, 50, 70, 90, 96% ethanol for 10 min each, followed by 4 × 10 min in 100% ethanol. The samples were then subjected to critical point drying and sputter-coated with a palladium–gold thin film before examination by SEM system (Zeiss) at 15 kV.

### Statistical analysis and data representation

All quantifications are representative of three independent experiments. The statistical analysis and graphical representations for all data were performed with R Studio (Version 1.0.15).

### Reporting summary

Further information on experimental design is available in the Nature Research Reporting Summary linked to this paper.

## Supplementary information

Supplementary Information

Reporting Summary

## Data Availability

All the data supporting the present findings are included in this article and in its supplementary information files. Additional scanning electronic microscopy photographs have been deposited at the Cell Image Library repository and are available at the following URLs: http://cellimagelibrary.org/groups/53296, http://cellimagelibrary.org/groups/53299. The datasets generated in the present study are available upon request to the corresponding author.

## References

[1] Kloos WE (1980). Natural populations of the genus *Staphylococcus*. Annu. Rev. Microbiol..

[2] Kim MW, Greenfield BK, Snyder RE, Steinmaus CM, Riley LW (2018). The association between community-associated *Staphylococcus aureus* colonization and disease: a meta-analysis. BMC Infect. Dis..

[3] Eriksen KR (1961). “Celbenin”-resistant staphylococci. Ugeskr Laeger.

[4] Jevons MP, Coe AW, Parker MT (1963). Methicillin resistance in staphylococci. Lancet.

[5] Fowler VG (2005). *Staphylococcus aureus* endocarditis: a consequence of medical progress. J. Am. Med. Assoc..

[6] Chambers HF, Deleo FR (2009). Waves of resistance: *Staphylococcus aureus* in the antibiotic era. Nat. Rev. Microbiol..

[7] DeLeo FR, Otto M, Kreiswirth BN, Chambers HF (2010). Community-associated meticillin-resistant *Staphylococcus aureus*. Lancet.

[8] Frank AL, Marcinak JF, Mangat PD, Schreckenberger PC (1999). Increase in community-acquired methicillin-resistant *Staphylococcus aureus* in children. Clin. Infect. Dis..

[9] Fridkin SK (2005). Methicillin-resistant *Staphylococcus aureus* disease in three communities. N. Engl. J. Med..

[10] Herold BC (1998). Community-acquired methicillin-resistant *Staphylococcus aureus* in children with no identified predisposing risk. J. Am. Med. Assoc..

[11] Kaplan SL (2005). Three-year surveillance of community-acquired *Staphylococcus aureus* infections in children. Clin. Infect. Dis..

[12] Otto M (2010). Basis of virulence in community-associated methicillin-resistant *Staphylococcus aureus*. Annu. Rev. Microbiol..

[13] Alzomor O, Alfawaz T, Alshahrani D (2017). Invasive community-acquired methicillin-resistant *Staphylococcus aureus* (CA-MRSA) infection in children: case series and literature review. Int. J. Pediatr. Adolesc. Med..

[14] Tong SY, Davis JS, Eichenberger E, Holland TL, Fowler VG (2015). *Staphylococcus aureus* infections: epidemiology, pathophysiology, clinical manifestations, and management. Clin. Microbiol. Rev..

[15] Ellington JK (2003). Intracellular *Staphylococcus aureus*. A mechanism for the indolence of osteomyelitis. J. Bone Jt. Surg. Br..

[16] Reygaert WC (2018). An overview of the antimicrobial resistance mechanisms of bacteria. AIMS Microbiol..

[17] Miragaia M (2018). Factors contributing to the evolution of *mecA*-mediated beta-lactam resistance in staphylococci: update and new insights from whole genome sequencing (WGS). Front. Microbiol..

[18] Peacock SJ, Paterson GK (2015). Mechanisms of methicillin resistance in *Staphylococcus aureus*. Annu. Rev. Biochem..

[19] Costerton JW (1987). Bacterial biofilms in nature and disease. Annu. Rev. Microbiol..

[20] Hall-Stoodley L, Costerton JW, Stoodley P (2004). Bacterial biofilms: from the natural environment to infectious diseases. Nat. Rev. Microbiol..

[21] Ito A, Taniuchi A, May T, Kawata K, Okabe S (2009). Increased antibiotic resistance of *Escherichia coli* in mature biofilms. Appl. Environ. Microbiol..

[22] Mah TF, O’Toole GA (2001). Mechanisms of biofilm resistance to antimicrobial agents. Trends Microbiol..

[23] Jamal M (2018). Bacterial biofilm and associated infections. J. Chin. Med. Assoc..

[24] Costerton JW, Stewart PS, Greenberg EP (1999). Bacterial biofilms: a common cause of persistent infections. Science.

[25] Lynch AS, Robertson GT (2008). Bacterial and fungal biofilm infections. Annu. Rev. Med..

[26] Dufrene YF, Persat A (2020). Mechanomicrobiology: how bacteria sense and respond to forces. Nat. Rev. Microbiol..

[27] Moormeier DE, Bayles KW (2017). *Staphylococcus aureus* biofilm: a complex developmental organism. Mol. Microbiol..

[28] Paluch E, Rewak-Soroczynska J, Jedrusik I, Mazurkiewicz E, Jermakow K (2020). Prevention of biofilm formation by quorum quenching. Appl. Microbiol. Biotechnol..

[29] WHO. *Geneva* (2020).

[30] Algburi, A., Comito, N., Kashtanov, D., Dicks, L. M. T. & Chikindas, M. L. Control of biofilm formation: antibiotics and beyond. *Appl. Environ. Microbiol*. 10.1128/AEM.02508-16 (2017). 10.1128/AEM.02508-16 PMC5244297 27864170

[31] Mataraci E, Dosler S (2012). In vitro activities of antibiotics and antimicrobial cationic peptides alone and in combination against methicillin-resistant *Staphylococcus aureus* biofilms. Antimicrob. Agents Chemother..

[32] Mathur H (2018). Fighting biofilms with lantibiotics and other groups of bacteriocins. NPJ Biofilms Microbiomes.

[33] Chikindas ML, Weeks R, Drider D, Chistyakov VA, Dicks LM (2018). Functions and emerging applications of bacteriocins. Curr. Opin. Biotechnol..

[34] Cotter PD, Hill C, Ross RP (2005). Bacteriocins: developing innate immunity for food. Nat. Rev. Microbiol..

[35] Newstead, L. L., Varjonen, K., Nuttall, T. & Paterson, G. K. Staphylococcal-produced bacteriocins and antimicrobial peptides: their potential as alternative treatments for *Staphylococcus aureus* Infections. *Antibiotics.*10.3390/antibiotics9020040 (2020). 10.3390/antibiotics9020040 PMC7168290 31973108

[36] Bastos MC, Ceotto H, Coelho ML, Nascimento JS (2009). Staphylococcal antimicrobial peptides: relevant properties and potential biotechnological applications. Curr. Pharm. Biotechnol..

[37] Vijay Simha B, Sood SK, Kumariya R, Garsa AK (2012). Simple and rapid purification of pediocin PA-1 from *Pediococcus pentosaceous* NCDC 273 suitable for industrial application. Microbiol. Res..

[38] Silva CCG, Silva SPM, Ribeiro SC (2018). Application of bacteriocins and protective cultures in dairy food preservation. Front. Microbiol..

[39] Alvarez-Sieiro P, Montalban-Lopez M, Mu D, Kuipers OP (2016). Bacteriocins of lactic acid bacteria: extending the family. Appl. Microbiol. Biotechnol..

[40] Mehmeti I, Bytyqi H, Muji S, Nes IF, Diep DB (2017). The prevalence of *Listeria monocytogenes* and *Staphylococcus aureus* and their virulence genes in bulk tank milk in Kosovo. J. Infect. Dev. Ctries.

[41] Ovchinnikov KV (2016). Novel group of leaderless multipeptide bacteriocins from gram-positive bacteria. Appl. Environ. Microbiol..

[42] Chi H, Holo H (2018). Synergistic antimicrobial activity between the broad spectrum bacteriocin garvicin KS and nisin, farnesol and polymyxin B against gram-positive and gram-negative bacteria. Curr. Microbiol..

[43] Akasapu S, Hinds AB, Powell WC, Walczak MA (2019). Total synthesis of micrococcin P1 and thiocillin I enabled by Mo(vi) catalyst. Chem. Sci..

[44] Degiacomi G (2016). Micrococcin P1—a bactericidal thiopeptide active against *Mycobacterium tuberculosis*. Tuberculosis.

[45] Gronseth T (2017). Lugol’s solution eradicates *Staphylococcus aureus* biofilm in vitro. Int. J. Pediatr. Otorhinolaryngol..

[46] Moussa S. H., A-H A. A., Farouk A. Tetrazolium/formazan test as an efficient method to determine fungal chitosan antimicrobial activity. *J. Mycol*. 10.1155/2013/753692 (2013).

[47] Perez RH, Zendo T, Sonomoto K (2018). Circular and leaderless bacteriocins: biosynthesis, mode of action, applications, and prospects. Front. Microbiol..

[48] Towle KM, Vederas JC (2017). Structural features of many circular and leaderless bacteriocins are similar to those in saposins and saposin-like peptides. MedChemComm.

[49] Garcia-Fernandez E (2017). Membrane microdomain disassembly inhibits MRSA antibiotic resistance. Cell.

[50] Perichon B, Courvalin P (2009). *VanA*-type vancomycin-resistant *Staphylococcus aureus*. Antimicrob. Agents Chemother..

[51] Drago L (2016). Antibiofilm activity of sandblasted and laser-modified titanium against microorganisms isolated from peri-implantitis lesions. J. Chemother..

[52] Chin CY (2015). Global transcriptional analysis of *Burkholderia pseudomallei* high and low biofilm producers reveals insights into biofilm production and virulence. BMC Genomics.

[53] Kong C (2018). Suppression of *Staphylococcus aureus* biofilm formation and virulence by a benzimidazole derivative, UM-C162. Sci. Rep..

[54] Wu S, Huang F, Zhang H, Lei L (2019). *Staphylococcus aureus* biofilm organization modulated by YycFG two-component regulatory pathway. J. Orthop. Surg. Res..

[55] Zhang Y, Chen M, Bruner SD, Ding Y (2018). Heterologous production of microbial ribosomally synthesized and post-translationally modified peptides. Front. Microbiol..

[56] Ciufolini MA, Lefranc D (2010). Micrococcin P1: structure, biology and synthesis. Nat. Prod. Rep..

[57] Mikolajka A (2011). Differential effects of thiopeptide and orthosomycin antibiotics on translational GTPases. Chem. Biol..

[58] Otaka T, Kaji A (1974). Micrococcin: acceptor-site-specific inhibitor of protein synthesis. Eur. J. Biochem..

[59] Zheng Q (2015). Thiopeptide antibiotics exhibit a dual mode of action against intracellular pathogens by affecting both host and microbe. Chem. Biol..

[60] M., R. F. H. (2015). Antimicrobial resistance in clinically important biofilms. World J. Pharmacol..

[61] Sadovskaya I (2010). High-level antibiotic resistance in *Pseudomonas aeruginosa* biofilm: the *ndvB* gene is involved in the production of highly glycerol-phosphorylated beta-(1->3)-glucans, which bind aminoglycosides. Glycobiology.

[62] Ventola CL (2015). The antibiotic resistance crisis: part 1: causes and threats. P T.

[63] Marks LR, Clementi EA, Hakansson AP (2013). Sensitization of *Staphylococcus aureus* to methicillin and other antibiotics in vitro and in vivo in the presence of HAMLET. PLoS ONE.

[64] Lefranc D, Ciufolini MA (2009). Total synthesis and stereochemical assignment of micrococcin P1. Angew. Chem. Int. Ed. Engl..

[65] Pu Y, Ke Y, Bai F (2017). Active efflux in dormant bacterial cells—new insights into antibiotic persistence. Drug Resist. Updat.

[66] Wood TK, Knabel SJ, Kwan BW (2013). Bacterial persister cell formation and dormancy. Appl. Environ. Microbiol..

[67] Dawson CC, Intapa C, Jabra-Rizk MA (2011). “Persisters”: survival at the cellular level. PLoS Pathog..

[68] Dworkin J, Shah IM (2010). Exit from dormancy in microbial organisms. Nat. Rev. Microbiol..

[69] Marimon O (2016). An oxygen-sensitive toxin-antitoxin system. Nat. Commun..

[70] Latha T (2019). MRSA: the leading pathogen of orthopedic infection in a tertiary care hospital, South India. Afr. Health Sci..

[71] Vandana S, Raje M, Krishnasastry MV (1997). The role of the amino terminus in the kinetics and assembly of alpha-hemolysin of *Staphylococcus aureus*. J. Biol. Chem..

[72] Schaefler S, Perry W, Jones D (1979). Methicillin-resistant strains of *Staphylococcus aureus* phage type 92. Antimicrob. Agents Chemother..

[73] Hawiger J, Niewiarowski S, Gurewich V, Thomas DP (1970). Measurement of fibrinogen and fibrin degradation products in serum by staphylococcal clumping test. J. Lab. Clin. Med..

[74] Collee, J. G., Fraser A. G., Marmion B. P., Simmons A. *Mackie and McCartney Practical Microbiology* (Churchill Livingston, New York, 1996).

[75] Neu HC, Fu KP (1978). Synergy of azlocillin and mezlocillin combined with aminoglycoside antibiotics and cephalosporins. Antimicrob. Agents Chemother..

[76] Bhusal Y, Shiohira CM, Yamane N (2005). Determination of in vitro synergy when three antimicrobial agents are combined against *Mycobacterium tuberculosis*. Int. J. Antimicrob. Agents.

[77] Hudzicki, J. *Kirby-Bauer Disk Diffusion Susceptibility Test Protocol* (ASM Microbe Library, 2009).

[78] Stepanovic S (2007). Quantification of biofilm in microtiter plates: overview of testing conditions and practical recommendations for assessment of biofilm production by staphylococci. APMIS.

[79] Perez LM (2010). A new microtitre plate screening method for evaluating the viability of aerobic respiring bacteria in high surface biofilms. Lett. Appl. Microbiol..

