# Supplementary material for: A bacteriocin-based antimicrobial formulation to effectively disrupt the cell viability of methicillin-resistant Staphylococcus aureus (MRSA) biofilms
Source: NPJ Biofilms Microbiomes. 2020 Dec 2;6:58. doi: 10.1038/s41522-020-00166-4 (PMC7710749; doi:10.1038/s41522-020-00166-4)
Supplement: Supplementary file 1 — Supplementary Information [file 41522_2020_166_MOESM1_ESM.pdf]

Supplementary Figure 1

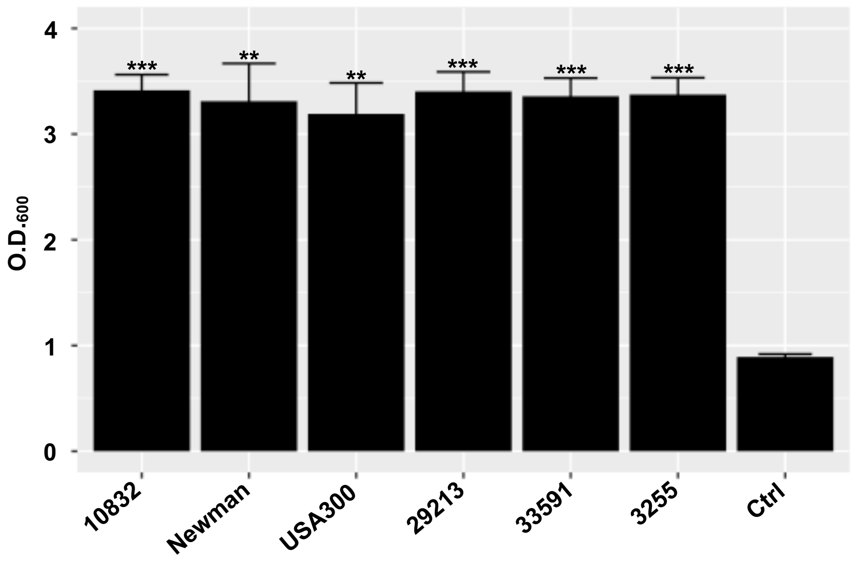

Supplementary Figure1. Biofilm formation ability of the selected *S. aureus* strains. Biofilms for the indicated strains were allowed to grow for 24h prior of being subjected to crystal violet staining. The amount of dye bound to the cells is an indirect measure of the biofilm-forming abilities and was quantified by optical density readings at 600 nm (O.D. 600) for each strain. The bar chart shows the average values (± s.d.) obtained from three independent experiments. The negative control (Ctrl) represents the average OD values measured for the control wells multiplied by 10. Asterisks within the bar chart represent the statistical significance (p-value) as determined by Welch´s t-test by comparing the average O.D. values obtained for each strain with the control (Ctrl). Asterisk representation of statistical significance: * $p \leq 0.05$; ** $p \leq 0.01$; *** $p \leq 0.001$; **** $p \leq 0.0001$.

Supplementary Figure 2

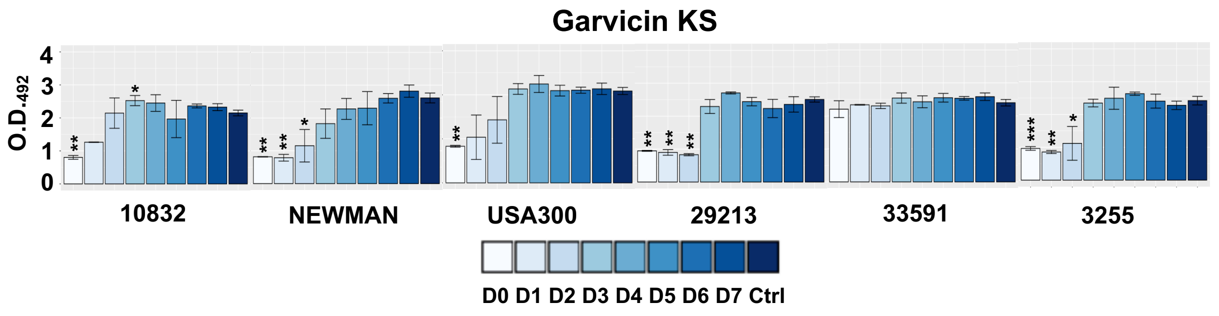

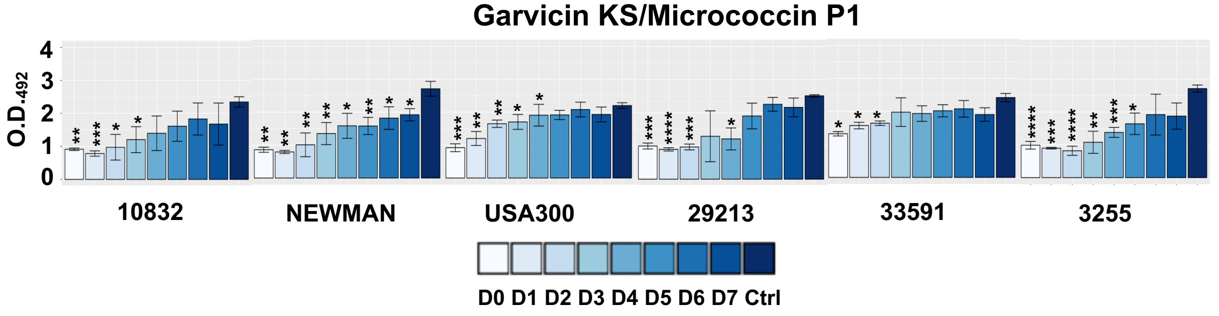

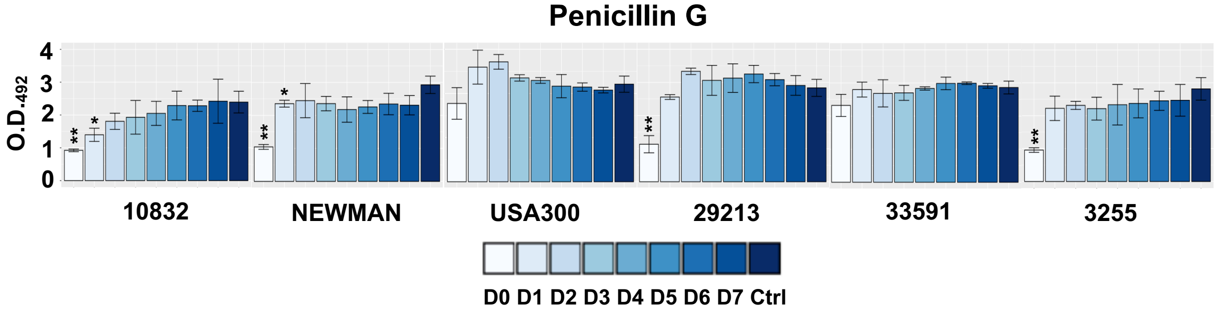

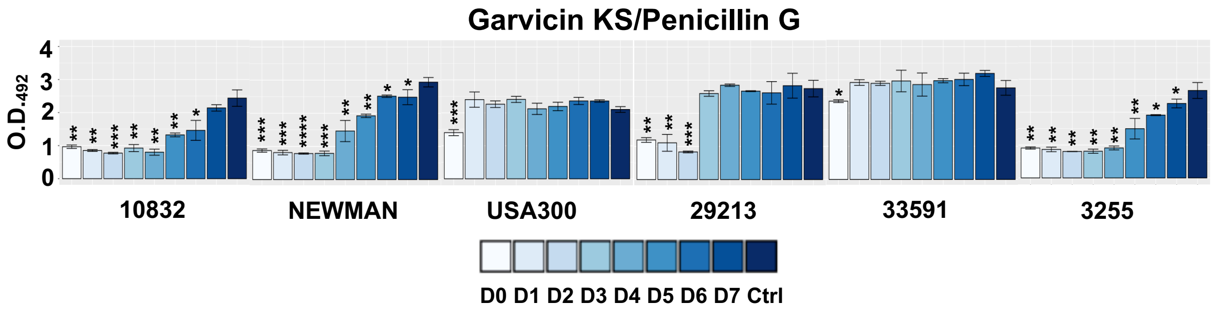

Supplementary Figure 2 continued

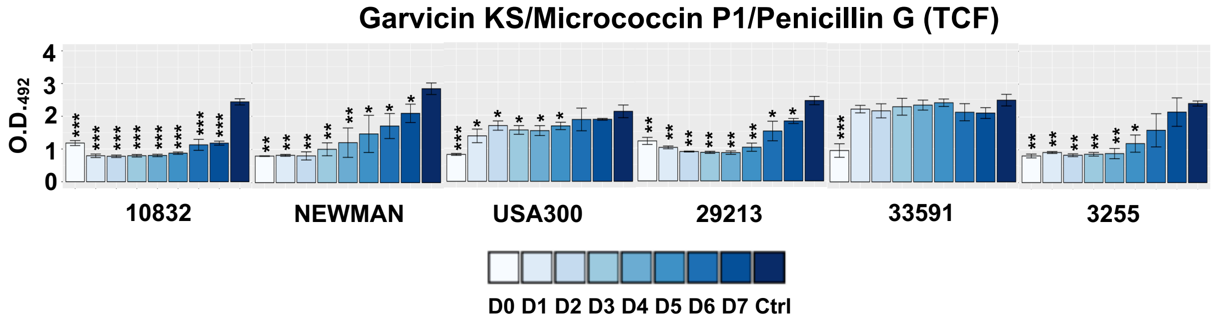

Supplementary Figure 2. Quantification of the bacterial metabolic activity following antimicrobial treatment of reference *S. aureus* strains. The quantification of the metabolic activity following BOAT assay with the indicated antimicrobials and their combinations was performed by optical density readings at 492 nm (O.D. 492). The average O.D. values (± s.d.) are shown in the histogram charts for each indicated antimicrobial. The control (Ctrl) values in the charts represent the averaged O.D. values obtained for the antimicrobial vehicle-treated strains. The concentration of the individual antimicrobials in the different dilutions (D0 to D7), and the relative control vehicles are described in figures 1 and 2. The data are representative of the average values obtained from three independent experiments. Asterisks within the bar chart represent the statistical significance (p-value) as determined by Welch´s t-test by comparing the average O.D. values obtained for each strain with the control (Ctrl). Asterisk representation of statistical significance: * $p \leq 0.05$; ** $p \leq 0.01$; *** $p \leq 0.001$; **** $p \leq 0.0001$.

Supplementary Figure 3

a

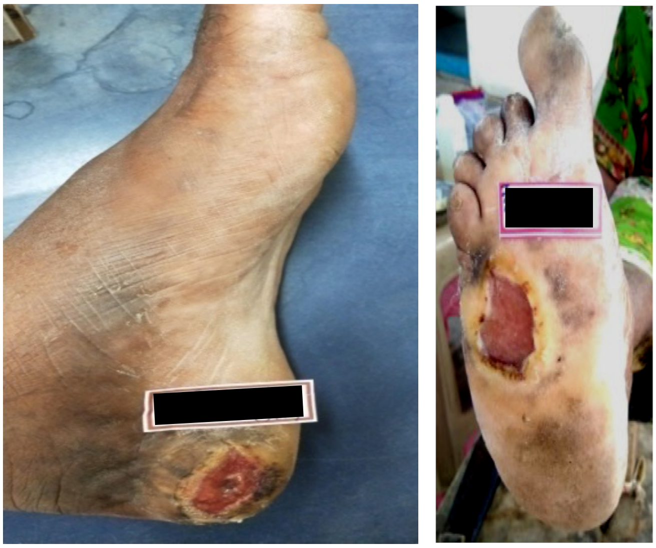

b

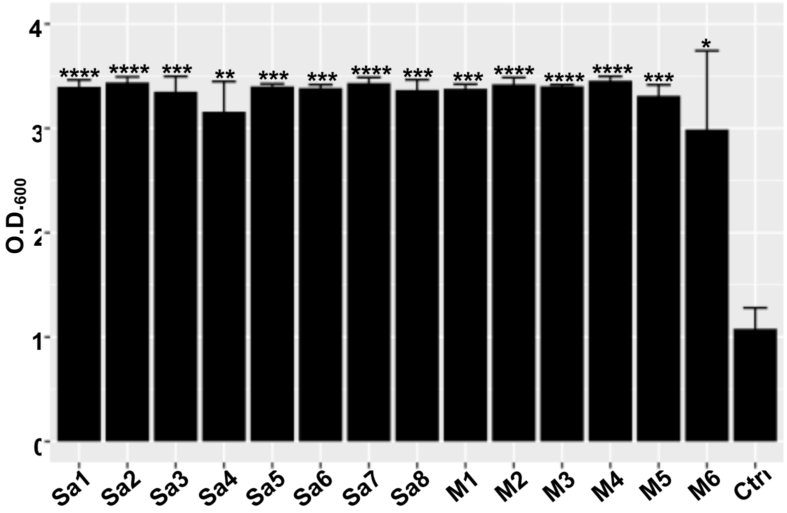

Supplementary Figure 3. The wound site and biofilm formation abilities of clinical *S. aureus* strains. a) Representative photographs of infected skin lesion from which the pathogenic strains were isolated. Photographs were published upon receipt of a written consent by the patients. b) Biofilms of the wound-associated *S. aureus* strains were allowed to grow for 24h prior of being subjected to crystal violet staining as detailed in supplementary figure 1A, and the bar chart shows the average values (± s.d.) obtained from three independent experiments. The assays were performed on 8 methicillin-sensitive (Sa1-8) and on 6 methicillin-resistant (M1-6) strains. The negative control (Ctrl) represents the average OD values measured for the control wells multiplied by 10. Asterisks within the bar chart represent the statistical significance (p-value) as determined by Welch´s t-test by comparing the average O.D. values obtained for each strain with the control (Ctrl). Asterisk representation of statistical significance: * $p \leq 0.05$; ** $p \leq 0.01$; *** $p \leq 0.001$; **** $p \leq 0.0001$.

Supplementary Figure 4

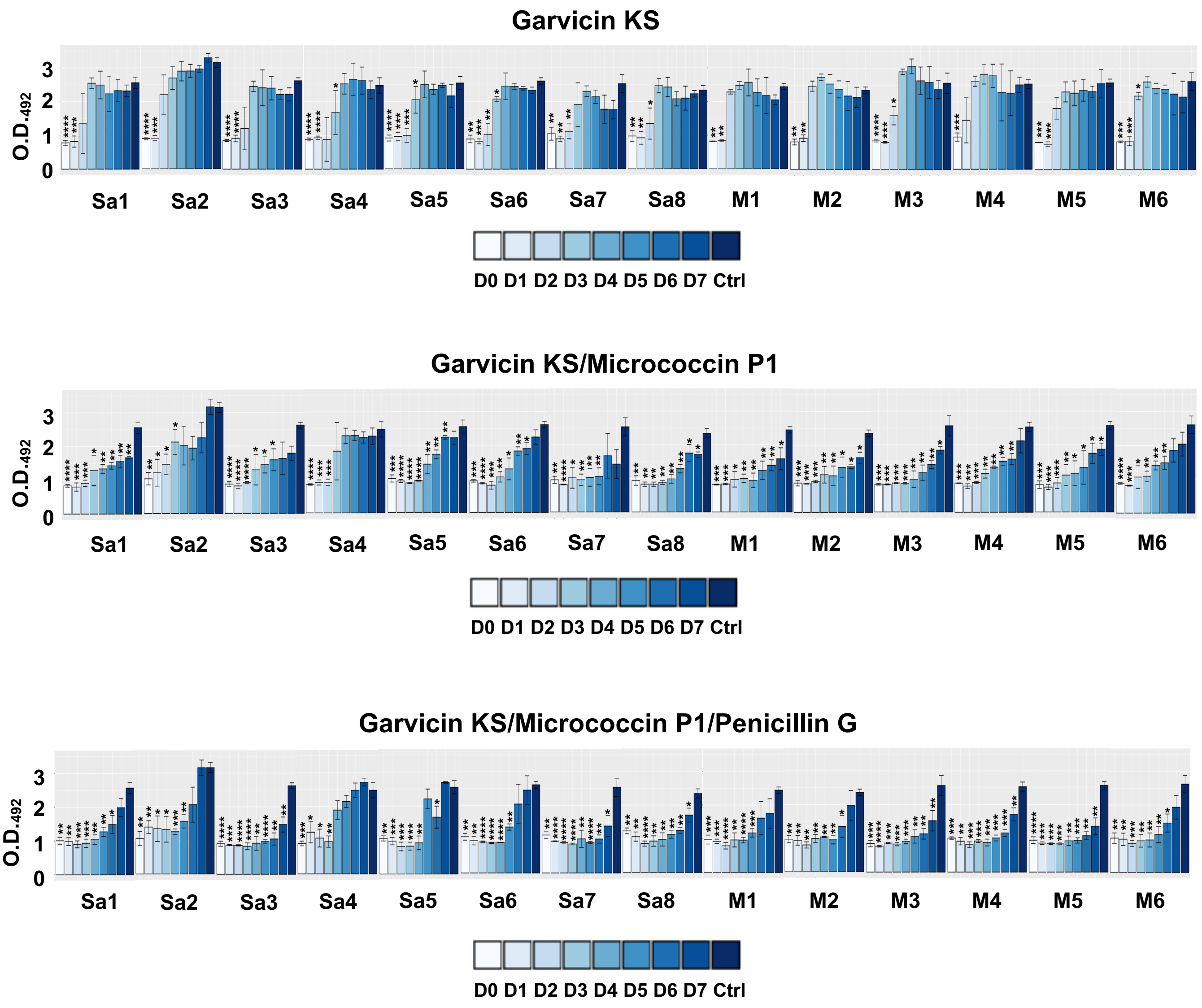

Supplementary figure 4. Quantification of the bacterial metabolic activity following antimicrobial treatment of clinical *S. aureus* strains. The histogram charts show the average levels (± s.d.) of the measured metabolic activity of clinical strains upon treatment with garvicin KS alone (GAK), in combination with micrococcin P1 (GAK/MP1) or with the tri-component formulation (TCF). The control (Ctrl) values in the charts represent the averaged O.D. values obtained for the antimicrobial vehicle-treated strains. The assays were performed on 8 methicillin-sensitive (Sa1-8) and on 6 methicillin-resistant (M1-6) strains. The concentration of the individual antimicrobials in the different dilutions (D0 to D7), and the relative control vehicles are described in figures 1 and 2. The data are representative of the average values obtained from three independent experiments. Asterisks within the bar chart represent the statistical significance (p-value) as determined by Welch´s t-test by comparing the average O.D. values obtained for each strain with the control (Ctrl). Asterisk representation of statistical significance: * p≤0.05; ** p≤0.01; *** p≤0.001; **** p≤0.0001.

Supplementary Table 1

Susceptibility of the reference *S. aureus* strains against the indicated antibiotics

Antibiotic[1] (μg)	ATCC 10832	NEWMAN	USA300	ATCC 29213	ATCC 33591	Sa3255
Cloxacillin (5)	S	S	R	S	R	S
Amoxicillin Clavulanic acid (30)	S	S	R	S	R	S
Penicillin-G (10)	S	S	R	S	R	S
Gentamicin (10)	S	S	R	S	S	S
Ciprofloxacin (5)	I	S	I	S	R	S
Levofloxacin (5)	S	S	R	S	R	S
Erythromycin (15)	S	S	R	S	R	S
Vancomycin (5)	S	S	S	S	S	S
Rifampicin (5)	R	R	S	S	R	R
Streptomycin (10)	S	S	R	S	R	S
Linezolid (10)	S	S	S	S	S	S

[1]The antibiotic susceptibilities (R – resistant; I – intermediate; S – sensitive) for the indicated strains were determined based on the inhibition zone diameters obtained around each antibiotic disc after a 24h incubation at 37°C, and in accordance with the CLSI interpretative standards. The concentration (in μg) for each of the indicated antibiotics is shown in brackets next to the antibiotic name. See the Methods section for details on the methodology.

Supplementary Table 2

Susceptibility of the clinical *S. aureus* isolates against the indicated antibiotics

Antibiotic[1]	Sa1	Sa2	Sa3	Sa4	Sa5	Sa6	Sa7	Sa8	M1	M2	M3	M4	M5	M6
Benzylpencillin	R	R	R	R	R	R	R	R	R	R	R	R	R	R
Oxacillin	S	S	S	S	S	S	S	S	R	R	R	R	R	R
Gentamicin	S	S	S	S	S	S	S	S	R	I	R	R	S	S
Ciprofloxacin	R	R	R	R	R	R	R	R	R	R	R	R	R	R
Levofloxacin	R	R	R	R	R	R	R	R	R	R	R	R	R	R
Clindamycin	S	S	S	S	S	S	S	S	S	R	S	S	S	S
Erythromycin	R	S	R	R	R	I	S	R	S	R	S	S	R	I
Vancomycin	S	S	S	S	S	S	S	S	S	S	S	S	S	S
Tetracycline	S	S	S	S	S	S	S	S	R	R	R	R	S	R
Tigecycline	S	S	S	S	S	S	S	S	S	S	S	S	S	S
Trimethoprim-Sulfamethoxazole	S	S	S	S	S	S	S	S	R	S	R	R	S	R
Linezolid	S	S	S	S	S	S	S	S	S	S	S	S	S	R

[1]Data on the antibiotic susceptibilities (R – resistant; I – intermediate; S – sensitive) for the indicated strains were analyzed and generated by the VITEK-2 system and its associated software. See the Methods section for details on the methodology.
