# Supplementary material for: A bacteriocin-based antimicrobial formulation to effectively disrupt the cell viability of methicillin-resistant Staphylococcus aureus (MRSA) biofilms
Source: NPJ Biofilms Microbiomes. 2020 Dec 2;6:58. doi: 10.1038/s41522-020-00166-4 (PMC7710749; doi:10.1038/s41522-020-00166-4)
Supplement: Supplementary file 2 — Reporting Summary [file 41522_2020_166_MOESM2_ESM.pdf]

nature research

Corresponding author(s): Prof. Dzung B. Diep

Last updated by author(s): Oct 19, 2020

Reporting Summary

Nature Research wishes to improve the reproducibility of the work that we publish. This form provides structure for consistency and transparency in reporting. For further information on Nature Research policies, see our Editorial Policies and the Editorial Policy Checklist.

Statistics

For all statistical analyses, confirm that the following items are present in the figure legend, table legend, main text, or Methods section.

n/a	Confirmed	
☐	☒	The exact sample size (*n*) for each experimental group/condition, given as a discrete number and unit of measurement
☐	☒	A statement on whether measurements were taken from distinct samples or whether the same sample was measured repeatedly
☐	☒	The statistical test(s) used AND whether they are one- or two-sided<br>*Only common tests should be described solely by name; describe more complex techniques in the Methods section.*
☐	☒	A description of all covariates tested
☐	☒	A description of any assumptions or corrections, such as tests of normality and adjustment for multiple comparisons
☐	☒	A full description of the statistical parameters including central tendency (e.g. means) or other basic estimates (e.g. regression coefficient) AND variation (e.g. standard deviation) or associated estimates of uncertainty (e.g. confidence intervals)
☐	☒	For null hypothesis testing, the test statistic (e.g. *F*, *t*, *r*) with confidence intervals, effect sizes, degrees of freedom and *P* value noted<br>*Give P values as exact values whenever suitable.*
☐	☒	For Bayesian analysis, information on the choice of priors and Markov chain Monte Carlo settings
☐	☒	For hierarchical and complex designs, identification of the appropriate level for tests and full reporting of outcomes
☐	☐	Estimates of effect sizes (e.g. Cohen's *d*, Pearson's *r*), indicating how they were calculated

Our web collection on statistics for biologists contains articles on many of the points above.

Software and code

Policy information about availability of computer code

Data collection	Data collection was performed with the following commercial softwares:<br>- SPECTROstar Nano v1.02 B1.H86<br>- Zeiss Zen Black v8.1.0.484<br>- SmartSEM v5.05
Data analysis	Data analysis was performed with the R studio software (v1.0.15)

For manuscripts utilizing custom algorithms or software that are central to the research but not yet described in published literature, software must be made available to editors and reviewers. We strongly encourage code deposition in a community repository (e.g. GitHub). See the Nature Research guidelines for submitting code & software for further information.

Data

Policy information about availability of data

All manuscripts must include a data availability statement. This statement should provide the following information, where applicable:

- Accession codes, unique identifiers, or web links for publicly available datasets
- A list of figures that have associated raw data
- A description of any restrictions on data availability

All the data supporting the present findings are included in this article and in its supplementary information files. The datasets generated in the present study are available upon request to the corresponding author.

Field-specific reporting

Please select the one below that is the best fit for your research. If you are not sure, read the appropriate sections before making your selection.

☒ Life sciences ☐ Behavioural & social sciences ☐ Ecological, evolutionary & environmental sciences

For a reference copy of the document with all sections, see nature.com/documents/nr-reporting-summary-flat.pdf

Life sciences study design

All studies must disclose on these points even when the disclosure is negative.

Sample size	*Describe how sample size was determined, detailing any statistical methods used to predetermine sample size OR if no sample-size calculation was performed, describe how sample sizes were chosen and provide a rationale for why these sample sizes are sufficient.*
Data exclusions	No data were excluded from the analysis
Replication	experiments were replicated independently at least three times. All attempts of replication were successful.
Randomization	All bacterial strains used in the study were divided in to ´control´ and ´treated´ groups.
Blinding	*Describe whether the investigators were blinded to group allocation during data collection and/or analysis. If blinding was not possible, describe why OR explain why blinding was not relevant to your study.*

Reporting for specific materials, systems and methods

We require information from authors about some types of materials, experimental systems and methods used in many studies. Here, indicate whether each material, system or method listed is relevant to your study. If you are not sure if a list item applies to your research, read the appropriate section before selecting a response.

Materials & experimental systems

n/a	Involved in the study	
☒	☐	Antibodies
☒	☐	Eukaryotic cell lines
☒	☐	Palaeontology and archaeology
☐	☒	Animals and other organisms
☐	☒	Human research participants
☒	☐	Clinical data
☒	☐	Dual use research of concern

Methods

n/a	Involved in the study	
☒	☐	ChIP-seq
☒	☐	Flow cytometry
☒	☐	MRI-based neuroimaging

Animals and other organisms

Policy information about studies involving animals; ARRIVE guidelines recommended for reporting animal research

Laboratory animals	The study did not involve laboratory animals.
Wild animals	The study did not involve wild animals.
Field-collected samples	Bacterial samples were collected from skin ulcers by using sterile pure viscose swabs. Each swab was inoculated on Blood and McConkey agar plates and bacterial isolates were allowed to grow overnight at 37 degrees Celsius.
Ethics oversight	The study did not involve laboratory animals and therefore an ethical approval for animal research is not applicable for the present study.

Note that full information on the approval of the study protocol must also be provided in the manuscript.

Human research participants

Policy information about studies involving human research participants

Population characteristics	Human research participants were enrolled in the study based on their previous diagnosis of Leprosy and on the presence of plantar ulcers from which bacterial strains were isolated.
Recruitment	Leprosy patients with plantar ulcers who were registered at LEPRA Society BPHRC, during December 2018 through May 2019

Recruitment	(n=31) were enrolled into the study after obtaining a written informed consent. No self-selection bias or other biases that may impact on the results are present.
Ethics oversight	The study protocol was approved by the Institutional Ethical Committee at LEPRA Society

Note that full information on the approval of the study protocol must also be provided in the manuscript.
